# Effects of a Lidocaine-Loaded Alginate/CMC/PEO Electrospun Nanofiber Film on Postoperative Pain and Peritoneal Adhesion in a Rat Model

**DOI:** 10.3390/medicina62040789

**Published:** 2026-04-20

**Authors:** Ha-young Kim, Hyo-jin Kim, Geun Joo Choi, Hyun Kang

**Affiliations:** 1 Department of Anesthesiology and Pain Medicine, College of Medicine, Chung-Ang University, Seoul 06911, Republic of Korea; 07692@cauhs.or.kr (H.-y.K.); raphaella@cau.ac.kr (H.-j.K.); 2 Department of Anesthesiology and Pain Medicine, Chung-Ang University Hospital, Seoul 06973, Republic of Korea; 3 Department of Anesthesiology and Pain Medicine, Chung-Ang University Gwangmyeong Hospital, Gwangmyeong 14353, Republic of Korea

**Keywords:** animal experimentation, electrospun nanofiber film, adhesion, inflammation, postoperative

## Abstract

*Background and Objectives*: Postoperative pain and intra-abdominal adhesions are common complications following surgery. Pain delays early mobilization, whereas adhesions can lead to bowel obstruction, chronic pain, or infertility. Current treatments, including systemic analgesics and physical barrier methods, are only partially effective. We hypothesized that combining these modalities would yield superior outcomes. Accordingly, we investigated whether a lidocaine-loaded alginate–carboxymethyl cellulose–polyethylene oxide (ACPE) electrospun film could more effectively reduce both postoperative pain and adhesion formation than either component alone. *Materials and Methods*: An electrospun nanofiber film composed of ACPE containing lidocaine was prepared. Its effects were evaluated in rats using an incisional pain and a peritoneal adhesion model. Four groups were compared: saline control, free lidocaine, drug-free ACPE film, and lidocaine-loaded ACPE film. Fifteen rats were allocated to each group. The primary outcome was the mechanical withdrawal threshold (MWT) after plantar incision, while secondary outcomes included histological changes and adhesion scores assessed by the Moreno system. *Results*: The lidocaine–ACPE film significantly increased MWT compared with all other groups, demonstrating a stronger and longer-lasting analgesic effect than free lidocaine. Adhesion scores were also lowest in the film group. Histological analysis confirmed a reduction in inflammatory cell infiltration and collagen deposition. *Conclusions*: A lidocaine-loaded ACPE nanofiber film effectively reduced both postoperative pain and adhesion formation in a rodent model. The combination of sustained local drug release and physical barrier function provides a promising strategy to address two major postoperative complications. Further preclinical studies are warranted before clinical application.

## 1. Introduction

Postoperative pain and intra-abdominal adhesions remain common complications that substantially affect postoperative recovery [1]. Incisional pain delays early mobilization, whereas adhesions may lead to bowel obstruction, chronic abdominal pain, infertility, or the need for reoperation [2,3,4,5]. Although multimodal analgesia and anti-adhesion barriers are widely employed, each approach has inherent limitations when used alone. Local anesthetics provide effective but short-lived analgesia, while physical barriers primarily prevent tissue apposition and adhesion recurrence [6,7,8,9]. Recent evidence suggests, however, that certain bioactive or polymeric barriers can also modulate inflammatory and fibrotic responses to surgical injury [10]. Therefore, an integrated strategy that addresses both pain control and adhesion prevention would be of considerable clinical value.

The electrospinning technique is a versatile and viable technique for generating ultrathin fibers [11]. This technique uses a high voltage to draw a polymer solution into ultrafine fibers, forming a nonwoven mat with high surface area, porosity, and distinctive microstructures [11,12]. Because of these advantageous features, electrospun films have emerged as promising anti-adhesion materials with excellent conformability to tissue surfaces [12,13]. Among these materials, alginate–carboxymethyl cellulose–polyethylene oxide (ACPE) composites are biocompatible and have been investigated as physical barriers against postoperative adhesions [14]. Concurrently, lidocaine is well known for its anesthetic properties and also exhibits anti-inflammatory activity by reducing neurogenic inflammation and peripheral sensitization [8,15]. Since postoperative adhesion formation is initiated by early inflammatory cascades, lidocaine may act as a chemical barrier that attenuates these inflammatory responses while providing analgesia [15,16]. Meanwhile, the ACPE electrospun nanofiber film serves as a mechanical barrier that prevents tissue apposition and early fibrin bridging [17,18]. This combined chemical–mechanical strategy may therefore improve anti-adhesion efficacy while simultaneously delivering sustained local analgesia.

Based on this rationale, we developed a lidocaine-loaded ACPE nanofiber film as a dual-purpose barrier designed to alleviate postoperative pain and prevent tissue adhesion.

We hypothesized that sustained lidocaine release from the nanofiber matrix would reduce postoperative hyperalgesia and function as a modest chemical barrier by attenuating early inflammatory responses, while the ACPE scaffold would inhibit fibroinflammatory activity and collagen deposition that promote adhesion formation. To test this hypothesis, we employed rodent models of incisional pain and peritoneal adhesion, comparing four interventions: saline control, free lidocaine, drug-free ACPE film, and lidocaine-loaded ACPE film containing the same dose of lidocaine as the free-drug group. Furthermore, a poloxamer/alginate/CaCl_2_ mixture was used as the reference anti-adhesive agent to compare the drug-free ACPE film and lidocaine-loaded ACPE film groups with a positive control group.

The primary endpoint was the mechanical withdrawal threshold (MWT) following plantar incision, which was used to assess analgesic efficacy. Secondary outcome measures included histological evaluation of inflammation and fibrosis, as well as macroscopic assessment of peritoneal adhesions using the Moreno scoring system. We anticipated that the lidocaine-loaded ACPE film would exhibit superior efficacy compared with either individual component, supporting its potential as an integrated therapeutic strategy.

## 2. Materials and Methods

### 2.1. Protocol and Registration

This randomized controlled experimental study was conducted in accordance with the Guide for the Care and Use of Laboratory Animals published by the National Institutes of Health (USA) and complied with the Animal Research: Reporting of In Vivo Experiments (ARRIVE) guidelines [19].

### 2.2. Preparation of Study Materials

ACPE electrospun nanofiber film and lidocaine-loaded ACPE were prepared using previously described methods [20]. A multi-nozzle electrospinning system was used to fabricate composite nanofibers by co-spinning polycaprolactone (PCL) and carboxymethyl cellulose (CMC). Lidocaine was incorporated into the nanofiber matrix to enable controlled drug release. For in vitro evaluation of anti-adhesive properties, the electrospun film was transferred onto cell culture dishes, and cells were seeded onto the film surface. To evaluate drug release behavior, the release profile of lidocaine from the nanofiber film was analyzed over time using high-performance liquid chromatography. Commercial 2% Lidocaine-HCl was purchased from Hanmi Pharmaceutical Co., Ltd. (Seoul, Republic of Korea), and a poloxamer/alginate/CaCl_2_ mixture was obtained from Genewel Co., Ltd. (Seongnam-si, Gyeonggi-do, Republic of Korea). All materials were used as received.

### 2.3. Animal Preparation

The experimental protocols were reviewed and approved by the Institutional Animal Care and Use Committee of Chung-Ang University (Approval No. 2018-00092). All experiments were performed in the Animal Research Laboratory of Chung-Ang University.

Adult male Sprague–Dawley rats (250–300 g; Coretec Laboratories, Seoul, Republic of Korea) were used in this study. Prior to the experiments, the animals were acclimated to the housing environment for one week. The rats were housed in pairs per cage under controlled temperature conditions (22 ± 0.5 °C) with a 12 h light/12 h dark cycle. Food and water were provided ad libitum throughout the study period. Female rats were excluded because fluctuations in hormone levels can affect pain thresholds [21]. Rats exhibiting any abnormalities were also excluded.

### 2.4. Randomization and Study Model

Participants were randomly allocated into four groups using a computer-generated randomization table based on Wei’s urn model, created with PASS™ 11 software (NCSS, Kaysville, UT, USA). A statistician who was not involved in the conduct of the study generated the randomization sequence. The details of the sequence were concealed from the investigators, and the group assignments were stored in sealed envelopes labeled only with the case numbers. To maintain investigator blinding, intra-plantar application and intraperitoneal injection were performed by an independent investigator with access to the allocation record. The intra-plantar preparations were loaded into 1 mL syringes containing 0.2 mL of normal saline or the study drug, while intraperitoneal preparations were loaded into 5 mL syringes containing 2 mL of normal saline or the study drug. For groups receiving ACPE or lidocaine-loaded ACPE, the electrospun films were pre-cut into standardized pieces and supplied in opaque containers to conceal their appearance prior to intraperitoneal administration. Because saline or lidocaine were administered as liquids, whereas ACPE and lidocaine-loaded ACPE were delivered as solid film constructs, complete masking of the physical form was not feasible. Nevertheless, all procedures involving administration, sample handling, and outcome assessment were conducted by separate investigators to minimize potential bias. Personnel responsible for behavioral or macroscopic evaluations remained blinded to group assignment throughout the study.

The experimental groups were treated as follows and 15 rats were allocated to each group:

Control group (Group C): Received 0.9% NaCl solution.

Lidocaine group (Group L): Received 2% lidocaine.

ACPE group (Group P): Received alginate/carboxymethyl cellulose/polyethylene oxide electrospun nanofiber film.

Lidocaine-loaded ACPE group (Group LP): Received lidocaine-loaded alginate/carboxymethyl cellulose/polyethylene oxide electrospun nanofiber film (containing the same lidocaine dose as Group L).

Poloxamer/alginate/CaCl_2_ mixture group (Group PAC): Received poloxamer/alginate/CaCl_2_ mixture (5 mL or 0.2 mL administered intraperitoneally or intra-plantarly, respectively).

### 2.5. Anesthesia and Surgical Procedure

All surgical procedures were performed by a single team under semisterile conditions, using only powder-free gloves. Rats were administered general anesthesia. Induction was achieved with 6% sevoflurane in 100% oxygen inside a sealed, transparent plastic chamber until the animals were immobile. For maintenance, a nonrebreathing anesthetic circuit mask delivered 1–3% sevoflurane in 100% oxygen throughout the surgery to ensure adequate analgesia. Cefazolin (20 mg/kg; Chong Kun Dang Pharmaceutical Co.,Seoul, Republic of Korea) was administered subcutaneously before the incision.

The incisional pain model was developed as previously described [22,23], with slight modifications to the reported technique. Briefly, after aseptic preparation of the left hind paw, a 1 cm longitudinal incision was made on the plantar surface using a surgical blade, beginning approximately 0.5 cm distal to the tibiotarsal joint and extending toward the digits. The plantaris muscle was then exposed, gently elevated, and incised along its longitudinal axis. Study drugs prepared in syringes and pre-cut nanofiber films were applied to the incision site according to group allocation, with the films placed directly over the desiccated area. The incision was closed with two horizontal mattress sutures using 5-0 nylon (Blue nylon 5-0; Ailee Co., Seoul, Republic of Korea). All rats were allowed to recover, and sutures were removed on postoperative day 3.

The peritoneal adhesion model was established as previously described [24], with minor modifications to the reported technique. Briefly, after shaving the abdominal skin and disinfecting it with povidone-iodine, a laparotomy was performed through a 4–5 cm midline incision beginning just below the umbilicus. The small bowel and cecum were carefully exteriorized from the peritoneal cavity. Adhesions were induced on the antimesenteric surfaces of both the cecum and small bowel. To ensure a consistent degree of tissue trauma, the surfaces were gently abraded without pressure using an electrocautery cleaner (Johnson & Johnson, New Brunswick, NJ, USA) until erosive bleeding appeared. All procedures were performed by a single investigator and monitored by two independent observers. Following abrasion, the assigned study treatment (saline, lidocaine, or pre-cut ACPE film) was applied directly to the injured peritoneal surfaces according to group allocation. The abdominal wall was then closed with a continuous 4-0 Vicryl suture, and numbered tail tags were used for identification.

### 2.6. Behavioral Measurements

Behavioral assessments were conducted using an incisional pain model. Each rat was placed on an elevated plastic mesh platform with 8 × 8 mm openings and enclosed beneath an inverted transparent plastic cage (21 × 27 × 15 cm) for a 15 min acclimation period. Following habituation, MWTs were evaluated using von Frey filaments (Stoelting Co., Wood Dale, IL, USA). The filaments were applied perpendicularly to the plantar surface of the hind paw, exerting just enough pressure to cause a slight bend. Filaments producing bending forces of 4, 9, 20, 59, 78, 98, 147, and 254 mN were tested sequentially until a withdrawal response occurred or the maximum force of 254 mN (cutoff value) was reached. Each filament was applied three times with 3 min intervals between applications. The lowest bending force that elicited a withdrawal response was defined as the MWT. A complete lift of the plantar surface away from the mesh platform was recorded as a positive withdrawal response, whereas partial lifting, ambulation, hunching, stretching, or licking behaviors were not considered withdrawals. Once a withdrawal response was observed, additional filaments with both higher and lower forces were tested to confirm the MWT. All MWT measurements were performed by a trained investigator blinded to group assignments. Assessments were conducted at the following time points: baseline, 1, 2, 4, 6, 8, 24, 48 h, and 1 week after surgery.

### 2.7. Macroscopic Assessment and Adhesion Score

In the intraperitoneal adhesion model, macroscopic adhesion assessment was performed. On the 14th postoperative day, animals were euthanized with CO_2_, and a U-shaped laparotomy—sparing the previous incision site—was conducted to examine all adhesions. Two referees, blinded to the study objectives, independently scored the adhesions, and a consensus score was assigned to each rat. A previously validated adhesion scoring system was applied to evaluate the number, location, thickness, tension, and vascularization of intra-abdominal adhesions. A total integration value (Moreno score) was calculated (Supplementary Table S1) [25,26]. The Moreno score is a validated, macroscopic assessment system used in animal studies to evaluate the severity of abdominal adhesions following the surgery. It quantifies adhesions by analyzing factors such as the number, location, thickness, vascularization, and tension of adhesion bands [26].

### 2.8. Microscopic and Histologic Assessment

Microscopic and histologic assessments were performed for both the incisional pain model and the intraperitoneal adhesion model. In the incisional pain model, plantar tissue samples were collected from the incision site, including the inflamed and abraded tissue approximately 1 mm on either side of the incision, using sterile disposable biopsy punches (Miltex, Bethpage, NY, USA). In the intraperitoneal adhesion model, all visceral organ specimens containing adhesions were excised. All harvested tissues were fixed in 10% neutral-buffered formalin for 24 h, embedded in paraffin, and sectioned at a thickness of 5 μm. The tissue sections were stained with hematoxylin and eosin according to the manufacturer’s protocol, using the avidin–biotin complex staining method.

A pathologist blinded to the experimental objectives, materials, and group assignments evaluated all tissue slides and assessed the degree of inflammation and fibrosis using a light microscope. Both parameters were scored from 0 to 4 according to modified, previously established grading systems [27,28]. The inflammatory response included lymphocytes, plasma cells, histiocytes, and polymorphonuclear leukocytes. The grading criteria were defined as follows: 0 = no inflammation; 1 = a few lymphocytes and plasma cells; 2 = mild inflammatory infiltrate composed of lymphocytes, plasma cells, and polymorphonuclear leukocytes; 3 = grade 2 plus neutrophils; and 4 = high concentrations (aggregations) of lymphocytes, plasma cells, polymorphonuclear leukocytes, histiocytes, and ulceration. Fibrosis was scored on a scale of 0 to 4 based on the extent of collagen deposition as follows: 0 = no fibrosis; 1 = mild fibrotic reaction around the wound; 2 = easily detected thick collagen bands; 3 = well-developed, dense collagen bands; and 4 = severe fibrotic response replacing large tissue areas.

### 2.9. Comparison of ACPE and Lidocaine-Loaded ACPE with Positive Control

A poloxamer/alginate/CaCl_2_ mixture was used as the reference anti-adhesive agent (group PAC) to compare group P and group LP with the positive control. Either 5 mL or 0.2 mL of the poloxamer/alginate/CaCl_2_ mixture was administered intraperitoneally or intra-plantarly.

### 2.10. Sample Size Calculation

The primary outcome measure for the incisional pain model was the MWT stimulus determined using von Frey filaments. To estimate the appropriate group size for evaluating the antinociceptive activity of a lidocaine-loaded alginate/carboxymethyl cellulose/polyethylene oxide electrospun nanofiber film, a pilot study was conducted to measure MWT in six rats with the incisional pain model (group C). Because the MWT data from the pilot study did not satisfy the Shapiro–Wilk test for normality, the data were analyzed after natural logarithmic (ln) transformation. The mean ln-transformed MWT values at baseline, 1, 2, 4, 6, 8, 24, 48 h, and 2 weeks after surgery were 4.24, 1.76, 1.76, 1.69, 1.69, 1.62, 1.87, 2.12, and 2.53 ln (mN), respectively. The standard deviations of the ln-transformed MWT values ranged from 0.10 to 1.02, with an autocorrelation of 0.7 between adjacent measurements from the same individual. For the power analysis, we assumed that the autocorrelation structure could be adequately characterized by a first-order autoregressive model. To compare between-group differences, the Geisser–Greenhouse corrected F-test for repeated-measures analysis of variance (ANOVA) was planned. We aimed to detect 0, 20, and 20% increases in MWT in groups P, L, and PL, respectively, compared with group C. Consequently, the standard deviation was estimated to be 0.21, and the actual effect standard deviation was 0.40, yielding an effect size of 0.51. With an α = 0.05 and statistical power of 80%, the calculated sample size was 12 rats per group. Considering a potential 20% attrition rate, 15 rats were allocated to each group.

For the intraperitoneal adhesion model, the primary outcome measure was the intergroup difference in the macroscopic adhesion score (Moreno Score) assessed during postmortem observations. The required sample size was calculated based on data from a previous study [29], which reported a mean (± standard deviation) adhesion score of 11.2 ± 2.6 in the control group. Assuming an equal standard deviation for groups L, P, and LP, we planned to detect 10, 20, and 30% reductions in the Moreno Score in groups L, P, and LP, respectively, compared with group C. With α = 0.05 (two-tailed) and 80% power, the required sample size was 13 rats per group. Allowing for a 13% dropout rate, 15 rats were allocated to each group.

Sample size calculations were performed using PASS 11™ software (NCSS, Kaysville, UT, USA).

### 2.11. Statistical Analysis

The Shapiro–Wilk test was performed to assess the normality of the variables. As none of the data met the normality assumption, nonparametric tests were applied. Between-group comparisons were conducted using the Kruskal–Wallis test followed by the Bonferroni post hoc test for multiple comparisons (0.05/6 = 0.0083).

For serial data, because the MWT did not pass the Shapiro–Wilk test, a natural log transformation was applied. The log-transformed variables met the normality assumption; therefore, the data satisfied the requirement for parametric analysis. Repeated-measures ANOVA was used with “time” (baseline, 1, 2, 4, 6, 8, 24, 48 h, and 1 week after surgery) as the within-subjects factor and “group” (control, P, L, and LP) as the between-subjects factor.

Because Mauchly’s test of sphericity indicated that the assumption of sphericity was violated for the MWT test (χ^2^ [35] = 267.25, *p* < 0.001, Mauchly’s W = 0.007) and for the positive pain control (χ^2^ [35] = 119.76, *p* < 0.001, Mauchly’s W = 0.047), a one-way Wilks’ lambda multivariate analysis of variance (MANOVA) was performed. In this analysis, “group” (control, P, L, and LP) was treated as the independent factor, and MWT at each time point (baseline, 1, 2, 4, 6, 8, 24, 48 h, and 1 week post-surgery) as the dependent variable. To compare MWT values at individual time points, univariate ANOVA with Bonferroni correction (α = 0.05/9 = 0.0056) was applied.

When the assumption of homoscedasticity, as evaluated using Levene’s test, was violated, Welch’s ANOVA was used as an alternative to standard ANOVA. If either conventional or Welch’s ANOVA indicated significant group differences, Tukey’s post hoc test (for equal variances) or Tamhane’s T2 test (for unequal variances) was conducted to identify specific group differences.

All data are expressed as mean ± standard deviation (SD) and were analyzed using SPSS version 23.0 (IBM Corp., Armonk, NY, USA). A *p*-value ≤ 0.05 was considered statistically significant.

## 3. Results

### 3.1. Study Animals

All rats successfully completed the study. Throughout the experimental period, they exhibited normal grooming behavior and consumed food and water at typical levels. Aside from reduced weight-bearing on the incision site, their gait demonstrated no notable abnormalities. No animals showed postoperative wound complications.

### 3.2. Behavioral Response

The results of MANOVA indicated a statistically significant difference among groups [F(27, 140.83) = 2.478, *p* = 0.002; Wilks’ λ = 0.321, partial η^2^ = 0.315] (Supplementary Figure S1). The MWTs at baseline did not differ significantly between groups. MWTs in group LP were significantly increased from 1 h to 48 h after surgery compared with groups C and P. MWTs in group L were significantly increased from 1 h to 24 h compared with group C, and from 1 h to 8 h compared with group P. The linear mixed-effects model also revealed a statistically significant difference among groups [F (3, 497.38) = 65.354, *p* < 0.001], which was significant for groups LP and L (0.75 [0.61–0.89], *p* < 0.001; 0.51 [0.37–0.65], *p* < 0.001, respectively), compared with group C.

### 3.3. Gross Adhesion Score

In the intraperitoneal adhesion model, Group C exhibited the most severe adhesions across all variables, followed by Group L in the gross adhesion score. In Group LP, the number of adhesions, grade, thickness, and tension were significantly lower than those in Group C; moreover, the number of adhesions, thickness, and tension were also significantly lower than those in Group L. Group P demonstrated decreased values for the number of adhesions, grade, thickness, and tension compared with Group C.

**Figure 1:**
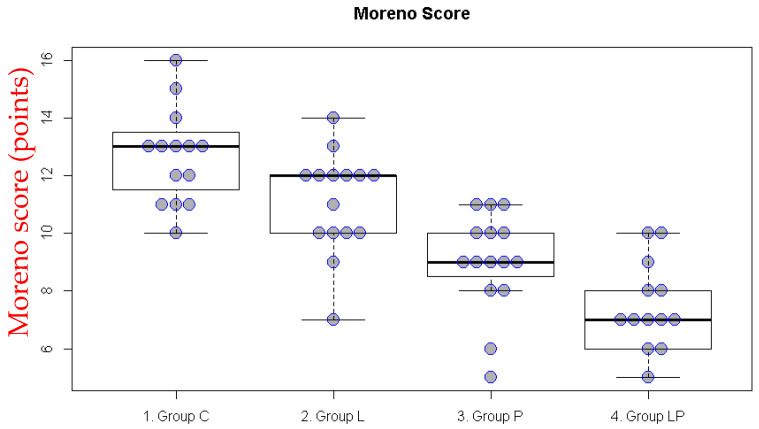
Gross adhesion scores in the intraperitoneal adhesion model. The boxplot illustrates the median and the first (Q1) and third (Q3) quartiles of the gross adhesion scores. Each dot represents the gross adhesion score for an individual rat.

Once all five subcategories were integrated, Group LP showed a significantly lower overall adhesion score than Groups C and L, whereas Group P exhibited a significantly lower score than Group C (Figure 1, Table 1).

### 3.4. Microscopic Evaluation

**Figure 2:**
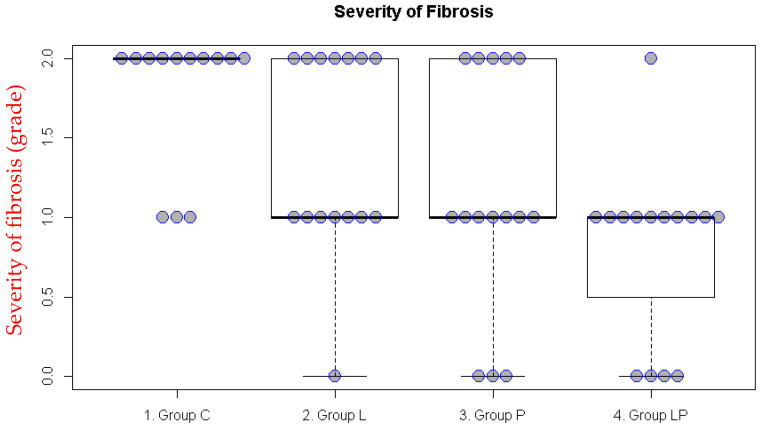
Fibrosis scores in the incisional pain model. The boxplot illustrates the median and the first (Q1) and third (Q3) quartiles of the fibrosis scores. Each dot represents the fibrosis score for an individual rat.

In the incisional pain model, significant differences were observed for inflammation and fibrosis (*p* < 0.001 and *p* = 0.003, respectively). The inflammation score was lower in Groups L and LP than in Group C (Supplementary Figure S2, Table 2), whereas the fibrosis score was lower in Groups P and LP than in Group C (Figure 2, Table 2).

**Figure 3:**
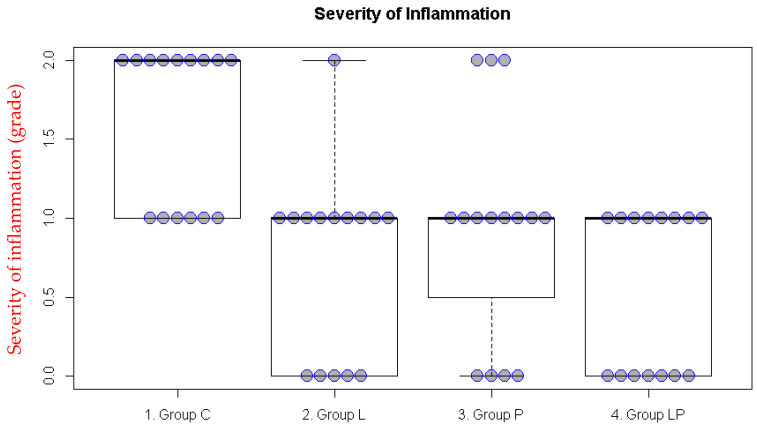
Inflammation scores in the intraperitoneal adhesion model. The boxplot illustrates the median and the first (Q1) and third (Q3) quartiles of the inflammation scores. Each dot represents the inflammation score for an individual rat.

**Figure 4:**
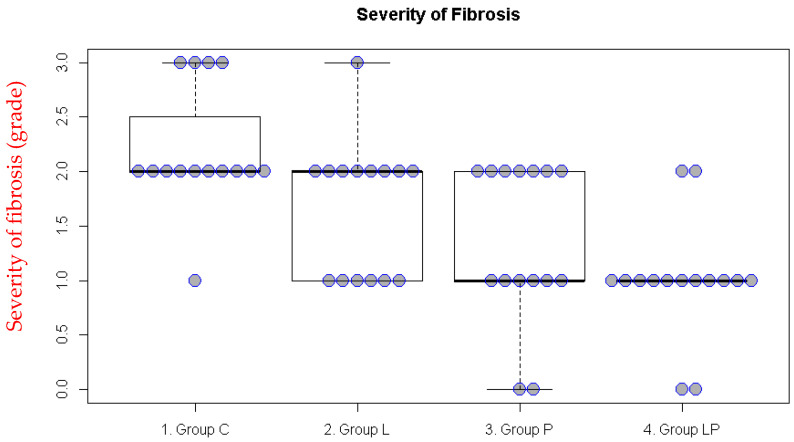
Fibrosis scores in the intraperitoneal adhesion model. The boxplot illustrates the median and the first (Q1) and third (Q3) quartiles of the fibrosis scores. Each dot represents the fibrosis score for an individual rat.

In the intraperitoneal adhesion model, significant differences were also observed for inflammation and fibrosis (both *p* < 0.001). The inflammation score was lower in Groups L and LP than in Group C (Figure 3, Table 2) while the fibrosis score was lower in Groups P and LP than in Group C (Figure 4, Table 2).

### 3.5. Comparison with Positive Control Group

In the incisional pain model, microscopic evaluations were performed in comparison with the positive control group. No evidence of differences was observed among group P, group LP, and group PAC with respect to inflammation and fibrosis (Table 2).

In the intraperitoneal adhesion model, gross adhesion scoring and microscopic evaluations were also performed with the positive control group. Compared with the positive control, both the number of adhesions and the Moreno score were lower in group LP than in groups P and PAC (Table 1). Furthermore, no evidence of differences was detected among group P, group LP, and group PAC in terms of inflammation and fibrosis (Table 2).

## 4. Discussion

This study demonstrates that a lidocaine-loaded ACPE electrospun nanofiber film exerts two significant effects in a surgical model: it alleviates postoperative pain and reduces intra-abdominal adhesion formation. The combined strategy outperformed both free lidocaine and the drug-free ACPE scaffold, supporting the hypothesis that physical and pharmacological approaches act more effectively when integrated.

The analgesic benefit observed in group LP is likely attributable to the sustained local release of lidocaine. Unlike the transient action of free lidocaine solution, incorporation into the nanofiber structure prolonged drug exposure at the wound site, thereby maintaining analgesic efficacy. This extended-release profile permitted continuous blockade of peripheral nociceptors, preventing the rebound sensitization commonly seen after administration of short-acting local anesthetics. In addition to sodium channel blockade, lidocaine possesses anti-inflammatory properties, which may have contributed to reduced peripheral sensitization and improved withdrawal thresholds in this model [15,30,31].

The scaffold itself served a distinct yet complementary function. Electrospun nanofibers provided a conformable surface capable of closely adhering to tissue, thereby limiting contact between injured serosa and preventing early fibrin deposition. The lower adhesion scores observed in the film group suggest that the combination of reduced inflammatory activity and a physical barrier created a microenvironment less conducive to adhesion formation. In particular, the sustained release of lidocaine likely contributed not only to analgesia but also to the suppression of early inflammatory cascades, which are key initiators of postoperative adhesion [32,33]. By attenuating these processes, lidocaine may have acted as a localized chemical barrier. This dual effect aligns with previous studies demonstrating the benefits of barrier materials and sustained-release anesthetic systems [34,35,36], although these modalities have rarely been integrated into a single platform. The structural stability of the ACPE nanofiber likely ensured both mechanical separation and prolonged drug retention, jointly mediating the observed synergy between analgesic and anti-adhesive outcomes [34,37,38]. Such sustained local exposure not only stabilized peripheral excitability but may also have modulated the inflammatory response in the surrounding tissue [32,33,39].

In addition to inflammation, the formation of fibrotic tissue is an important factor contributing to postoperative adhesions [40]. Fibrosis scores were lower in group P than in group C, supporting the hypothesis that applying a physical barrier before incision closure can reduce fibroblast proliferation and collagen deposition, thereby preventing adhesion formation [41,42]. Although the optimal coverage area of the barrier remains uncertain, these findings suggest that complete coverage of the traumatized site may maximize the anti-fibrotic effect.

Our previous study, which used a lidocaine-loaded poloxamer/alginate/CaCl_2_ mixture, evaluated inflammation and fibrosis histologically two weeks after surgery [16]. However, that formulation provided only short-term analgesia and partial attenuation of inflammatory infiltration, likely because of the rapid diffusion of lidocaine from the hydrogel matrix and the limited mechanical stability of the barrier. In contrast, the current ACPE nanofiber film preserved both the pharmacological and structural integrity of the barrier, allowing sustained local drug exposure during the critical inflammatory period. The present study incorporated quantitative histologic analysis and correlated microscopic findings with both behavioral and macroscopic outcomes. The markedly lower inflammatory cell infiltration observed in the lidocaine-loaded ACPE group suggests that prolonged lidocaine release not only reduced nociceptive sensitization but also suppressed inflammation and fibrosis at the wound site. These results expand upon our previous observations and strengthen the hypothesis that integrating chemical and physical barriers within a nanofibrous architecture enhances both analgesic and anti-adhesive efficacy.

These findings align with previous reports indicating that electrospun nanofiber-based scaffolds can sustain local drug concentrations and maintain prolonged release due to their high porosity and structural stability [34]. Conversely, hydrogel- or collagen-based barriers often permit rapid drug diffusion and loss of mechanical integrity, resulting in only transient analgesic effects [43,44]. The electrospun ACPE matrix developed in this study likely achieved superior drug retention and barrier stability, which contributed to its more durable analgesic and anti-adhesive performance [20].

The clinical implications of this material are noteworthy. Postoperative pain management and adhesion prevention are typically addressed as separate challenges; however, both processes originate from overlapping inflammatory pathways. A film capable of targeting both aims may decrease the need for systemic analgesics—particularly opioids—while simultaneously reducing the risk of adhesion-related complications such as bowel obstruction or infertility [3,4,45]. Moreover, the nanofiber platform provides flexibility for further modification, for instance, through the incorporation of anti-fibrotic or antimicrobial agents, which could broaden its potential applications [46].

This study has several limitations. It was conducted in a rodent model, and the findings may not fully translate to human surgery. The in vivo release kinetics of lidocaine were not characterized in detail, and potential systemic absorption or dose-dependent effects cannot be ruled out. Only one formulation and one dose were evaluated, indicating that optimization of the drug content and fiber properties remains necessary. Finally, long-term outcomes, such as effects on tissue healing, were not assessed and should be investigated in future studies.

## 5. Conclusions

In conclusion, the lidocaine-loaded ACPE electrospun film demonstrated both analgesic and anti-adhesive effects in an animal model. As shown in the results, the lidocaine-loaded ACPE electrospun film reduced postoperative pain and decreased adhesion formation in an animal model. These findings suggest that this approach may offer a dual therapeutic benefit by simultaneously addressing pain control and adhesion prevention. Although further preclinical research is warranted—particularly studies in larger animal models and using optimized formulations—the present findings indicate a promising strategy that may enhance both short- and long-term postoperative outcomes for surgical patients.

## Figures and Tables

**Table 1:** Gross adhesion scores in the intraperitoneal adhesion model: Main and positive control comparisons.

Comparison	Group	Number of Adhesions	Grade	Thickness	Tension	Vascularization	Moreno Score
Main comparison	Group C (n = 15)	3.80 ± 1.32	3.67 ± 0.72	2.53 ± 0.52	2.53 ± 0.52	0.47 ± 0.52	13.00 ± 2.10
	Group L (n = 15)	2.80 ± 0.56	3.27 ± 1.10	2.27 ± 0.70	2.27 ± 0.70	0.47 ± 0.52	11.07 ± 1.75
	Group P (n = 15)	2.40 ± 0.74 *	2.73 ± 0.80 *	1.73 ± 0.70 *	1.67 ± 0.72 *	0.40 ± 0.51	8.93 ± 1.87 *
	Group LP (n = 15)	1.40 ± 0.82 *^,†^	2.20 ± 1.15 *	1.27 ± 0.80 *^,†^	1.40 ± 0.74 *^,†^	0.20 ± 0.41	6.47± 2.97 *^,†^
	*p*-value	< 0.001	0.001	< 0.001	< 0.001	0.351	<0.001
Positive control comparison	Group P (n = 15)	2.40 ± 0.74	2.73 ± 0.80	1.73 ± 0.70	1.67 ± 0.72	0.40 ± 0.51	8.93 ± 1.87
	Group LP (n = 15)	1.40 ± 0.82 ^‡,§^	2.20 ± 1.15	1.27 ± 0.80	1.40 ± 0.74	0.20 ± 0.41	6.47 ± 2.97 ^‡,§^
	Group PAC (n = 15)	2.47 ± 0.74	2.73 ± 0.70	1.87 ± 0.52	1.80 ± 0.56	0.47 ± 0.52	9.33 ± 1.11
	*p*-value	0.002	0.351	0.039	0.336	0.228	0.002

* <0.05 compared with Group C; ^†^ <0.05 compared with Group L; ^‡^ <0.05 compared with Group P; ^§^ <0.05 compared with Group PAC. Data are presented as mean ± SD. The Kruskal–Wallis test was performed because of abnormal data distribution.

**Table 2:** Microscopic inflammation and fibrosis in the incisional pain and intraperitoneal adhesion models: Main and positive control comparisons.

		Incisional Pain Model		Intraperitoneal Adhesion Model	
		Inflammation	Fibrosis	Inflammation	Fibrosis
Main comparison	Group C (n = 15)	1.33 ± 0.62	1.93 ± 0.62	1.60 ± 0.51	2.20 ± 0.56
	Group L (n = 15)	0.60 ± 0.51 *	1.40 ± 0.63	0.73 ± 0.59 *	1.67 ± 0.62
	Group P (n = 15)	0.80 ± 0.68	1.13 ± 0.74 *	0.93 ± 0.70	1.33 ± 0.72 *
	Group LP (n = 15)	0.47 ± 0.52 *	0.80 ± 0.56 *	0.53 ± 0.52 *	1.00 ± 0.53 *
	*p*-value	0.003	<0.001	<0.001	<0.001
Positive control comparison	Group P (n = 15)	0.80 ± 0.68	1.13 ± 0.74	0.93 ± 0.70	1.33 ± 0.72
	Group LP (n = 15)	0.47 ± 0.52	0.80 ± 0.56	0.53 ± 0.52	1.00 ± 0.53
	Group PAC (n = 15)	0.73 ± 0.46	1.20 ± 0.68	1.07 ± 0.70	1.40 ± 1.06
	*p*-value	0.087	0.350	0.255	0.212

* <0.05 compared with Group C; Data are presented as mean ± SD. The Kruskal–Wallis test was used due to non-normal distribution.

## Data Availability

All data generated or analyzed during this study are included in this published article and its Supplementary Materials.

## Supplementary Materials

The following supporting information can be downloaded at: https://www.mdpi.com/article/10.3390/medicina62040789/s1.

## Abbreviations

The following abbreviations are used in this manuscript:

ACPE	Alginate–carboxymethyl cellulose–polyethylene oxide
MWT	Mechanical withdrawal threshold
