# Supplementary material for: Effects of a Lidocaine-Loaded Alginate/CMC/PEO Electrospun Nanofiber Film on Postoperative Pain and Peritoneal Adhesion in a Rat Model
Source: Medicina (Kaunas). 2026 Apr 20;62(4):789. doi: 10.3390/medicina62040789 (PMC13118005; doi:10.3390/medicina62040789)
Supplement: Supplementary file 1 [file medicina-62-00789-s001.zip › medicina-4220198--Supplementary information.pdf]

Supplementary Information

Supplementary Figures

Supplementary Figure S1. Mechanical withdrawal threshold(MWT) following surgery

Time-dependent changes in mechanical withdrawal threshold (MWT) in the control (C), placebo film (P), lidocaine solution (L), and lidocaine-loaded alginate–carboxymethyl-cellulose–polyethylene oxide (ACPE) film (LP) groups.

Data are presented as mean ± SEM (standard error of the mean).

* $P < 0.05$ vs C; † $P < 0.05$ vs P.

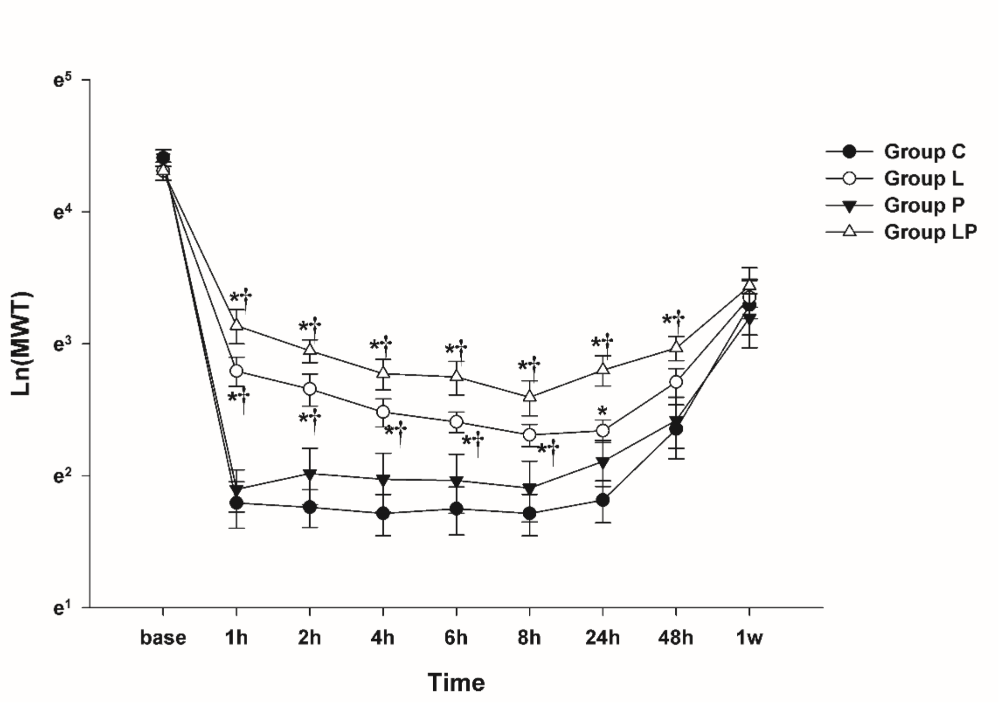

Supplementary Figure S2. Inflammation scores in the incisional model

Box-and-whisker plots showing histologic inflammation grades in each group.

Data are presented as median (IQR).

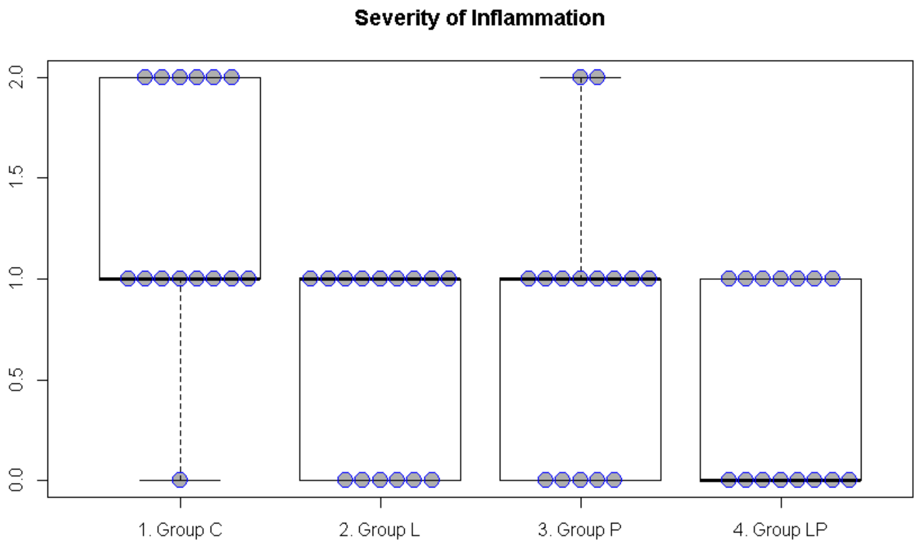

Supplementary Table S1. Criteria for gross adhesion scoring (Moreno system)

Criterion	Quantification score
Adhesions (n; site of adhesion)	1 per adhesion
Pelvic fat body-abdominal wall	1
Omentum-abdominal wall	2
Intestine-abdominal wall	3
Pelvic fat body or omentum-bowel	4
Intestine-intestine	5
Thickness	
<3 mm	1
3–5 mm	2
>5 mm	3
Tension	
Type I (loosening without dissection)	1
Type II (loosening with blunt dissection)	2
Type III (loosening with sharp dissection)	3
Vascularization	
Not vascularized	0
Vascularized	1
